# Extracellular vesicles isolated from frozen whole blood show biomarker potential in small intestine neuroendocrine neoplasms

**DOI:** 10.1016/j.isci.2026.116723

**Published:** 2026-07-14

**Authors:** Jonas Burman, Chuanwen Fan, Mohamed El Husseiny, Paulina Velasco Riestra, Alexander Sandberg, Constantinos P. Zambirinis, Róbert Kotán, Lúcia Amorim, Oliver Gimm, Linda Bojmar

**Affiliations:** 1 Department of Biomedical and Clinical Sciences, Linköping University, Linköping, Sweden; 2 Department of Medicine (Solna), Karolinska Institutet, Stockholm, Sweden; 3 Crown Princess Victoria Children’s Hospital, Region Östergötland, Linköping, Sweden; 4 Clinical Department of Surgery in Linköping, Region Östergötland, Linköping, Sweden; 5 Children’s Cancer and Blood Foundation Laboratories, Departments of Pediatrics, and Cell and Developmental Biology, Drukier Institute for Children’s Health, Meyer Cancer Center, Weill Cornell Medicine, New York, NY 10021, USA; 6 Science for Life Laboratory (SciLifeLab), Linköping University, Linköping, Sweden

**Keywords:** neuroendocrine neoplasms, extracellular vesicles, liquid biopsy, rare cancers, biobanks

## Abstract

Despite widespread storage of frozen whole blood (FWB) in biobanks worldwide, its suitability for extracellular vesicle (EV) research remains largely unassessed. Here, we developed a robust and practical workflow for EV-based cancer biomarker analysis from FWB by comparing differential ultracentrifugation (dUC), size-exclusion chromatography, and their combination. All methods successfully recovered vesicles within the expected 50–150 nm range, enriched for canonical EV markers, while reducing blood-derived EV contaminants, and displayed vesicle-like morphology. Among tested methods, dUC emerged as the most cost-effective and labor-efficient approach. Applying this pipeline to biobanked FWB samples from patients with small intestinal neuroendocrine neoplasms yielded an average of ∼1,000 proteins detected per sample using mass spectrometry-based proteomics, including known neuroendocrine markers and enrichment in synapse organization signaling pathways. These findings demonstrate the feasibility of EV isolation from FWB and biomarker-relevant downstream analysis, highlighting the untapped potential of EV-based biomarker discovery across diseases from existing biobanks.

## Highlights

• First study on extracellular vesicle (EV) isolation from frozen whole blood (FWB)
• Vesicles from FWB have expected size, morphology and marker expression of small EVs
• FWB EVs capture neuroendocrine neoplasm-related proteomic signatures in patients
• FWB EVs may be an efficient method for leveraging rare disease biobanked samples

## Graphical abstract

**Figure:**
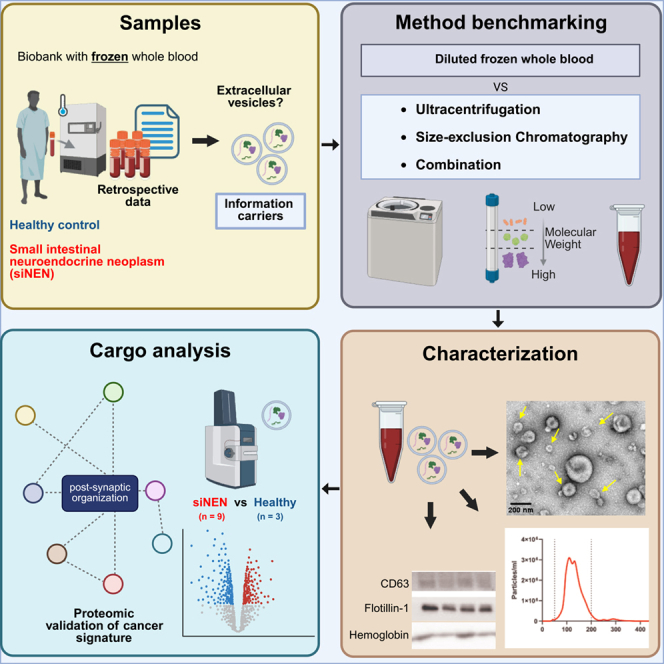

## Teaser

Oncology; Proteomics; extracellular vesicles

## Introduction

Biobanks serve as a cornerstone for modern biomedical research by systematically collecting and storing clinical samples alongside comprehensive follow-up data, creating a robust platform for investigating disease mechanisms, identifying biomarkers, and developing personalized disease management strategies.^1^ Unlike tissue biopsies, which offer a temporally static and spatially localized snapshot of diseased organs, blood has several advantages; it can be obtained through a minimally invasive venipuncture, it reflects the heterogeneity that occurs within diseased organs, and it can be sampled repeatedly over time, making it uniquely suited for longitudinal investigations of disease onset, progression, and treatment response.^2^ Storing blood has been implemented in several landmark cohort studies: between 2006 and 2010, the UK Biobank collected ethylenediaminetetraacetate (EDTA)-anticoagulated whole blood from 500,000 participants aged 40–69, along with plasma, serum, and buffy coat,^3^ the Norwegian HUNT study has archived over 120,000 whole blood samples across four survey waves (HUNT1–4),^4^ and although the US prostate, lung, colorectal, and ovarian cancer screening trial focused on serum, a substantial subset of its 150,000 participants had whole blood frozen during the third follow-ups.^5^ In the biobanks of low- and middle-income countries, where internationally standardized procedures for biobanking may be followed to a lesser extent, whole blood is commonly stored in freezers at −80°C.^6^ These frozen whole blood (FWB) collections represent an unprecedented resource for probing disease biology.

Although FWB is extensively banked, its broader use still faces significant technical challenges. For example, ice-crystal formation damages cell membranes and induces extensive hemolysis of red blood cells (RBCs), which outnumber white blood cells by roughly 1,000:1, releasing more than 100 mg/mL of hemoglobin (Hb) and intracellular RNA.^7^ Additionally, freeze-thaw cycles can alter plasma protein conformations, with at least 15% of proteins undergoing irreversible aggregation upon repeated thawing. Pre-analytical variability further complicates matters, for instance, EDTA- versus acid-citrate-dextrose-anticoagulated blood.^5^^,^^8^ Consequently, biobanked FWB is often used for DNA-based studies, while RNA, protein and other low-abundance analytes are less commonly analyzed, severely limiting its broader application in precision medicine.^9^

Extracellular vesicles (EVs) offer a promising way to overcome many of these limitations, as they can protect and preserve biomolecules within stable lipid bilayers. Although hemolysis resulting from freeze-thaw cycles increases RBC-derived EV subpopulations, optimized isolation workflows can remove most micron-sized debris and Hb contamination.^10^ Information for studies of EVs generally discourages the use of hemolyzed samples due to concerns about altered vesicle composition and purity. Nevertheless, it may also unveil novel biomarkers, as recent evidence indicates that RBC-derived microparticles and EVs can serve as highly accurate disease indicators, potentially enhanced by stress-induced functional changes.^11^ These findings raise the possibility that EVs recovered from FWB could provide insights not only into systemic or organ-specific alterations but also into stress-related functional states. However, existing EV-isolation protocols are tailored to fresh or thawed plasma and there has been no systematic evaluation of EV recovery or quality assessment from FWB.

We therefore aimed to address this methodological gap by developing and validating an optimized workflow for EV isolation from FWB, to demonstrate its feasibility and to harness the biomarker discovery potential of biobanked specimens. This workflow was systematically compared with existing EV-isolation protocols to establish a rapid, efficient, and scalable approach suitable for biobanked FWB samples. Its reproducibility and robustness were further validated using biobanked FWB samples from patients with small intestine neuroendocrine neoplasms (siNENs), a rare cancer with an annual incidence of approximately one per 100,000.^12^

## Results

### FWB has not been systematically investigated as a source of EVs

**Figure 1:**
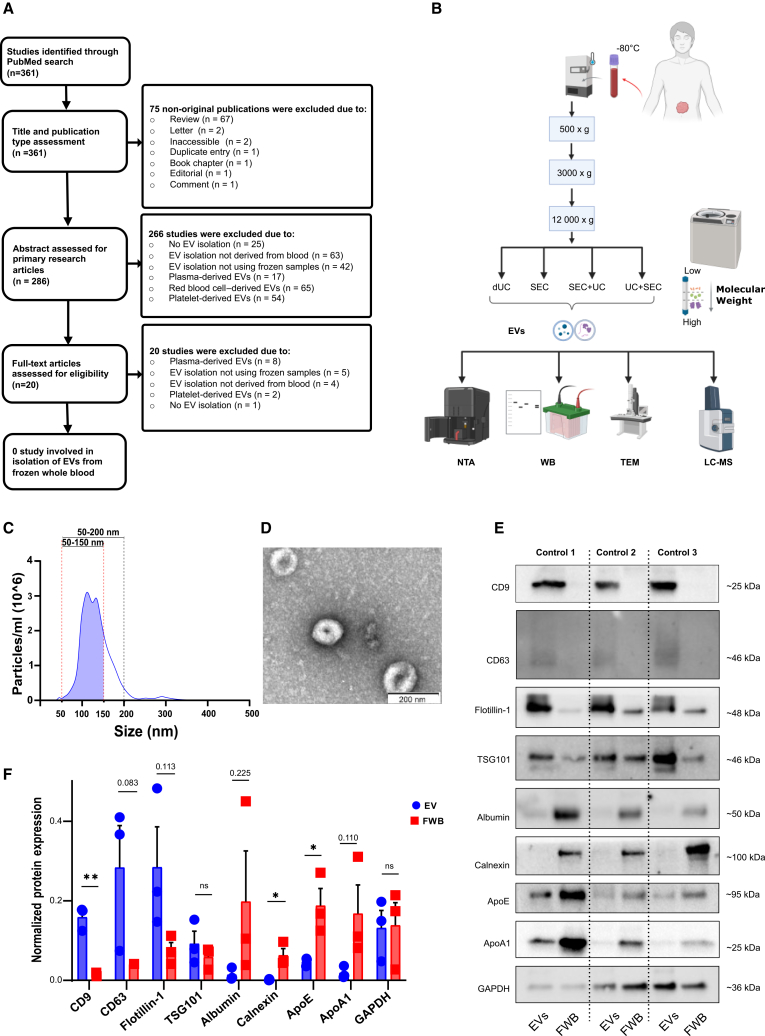
Characterization of EVs isolated from FWB (A) Schematic overview of literature search identifying gap in research of EV isolation from FWB. (B) Overview of protocols for isolation of EVs from healthy control and siNEN FWB by dUC and SEC. (C) Normalized particle size distribution of FWB EVs, measured by nano-particle tracking. (D) Representative electron microscopy image from FWB EVs, scale bar, 200 nm. (E) Representative immunoblotting results from FWB EVs and FWB. (F) Normalized protein expression for common EV and contamination markers. *n* = 3. ∗*p* < 0.05, ∗∗*p* < 0.01, ns not significant, by student’s *t* test. Error bars represent SEM.

To systematically evaluate whether FWB (including both cryopreserved and frozen blood) has been explored as a source for EVs isolation, we conducted a PubMed search on July 27^th^, 2025. This search retrieved a total of 361 entries, comprising 286 primary research articles, and 75 non-original publications based on title and publication type of screening (Figure 1A). Abstract screening further excluded 266 studies leaving 20 potentially relevant articles. However, full-text assessment showed that eight focused on plasma EVs, five investigated non-FWB, four used non-blood samples, two articles studied platelet-derived EVs, and one did not include EV isolation. Thus, no study was identified that specifically addressed EVs isolation from FWB (Figure 1A; Table S3). Based on these findings, we designed an experimental study to evaluate the feasibility of EV isolation from FWB, systematically compare isolation protocols, and assess their downstream applicability using samples from healthy donors and biobanked specimens of rare cancers such as siNENs (Figure 1B).

### EVs can be isolated from FWB

To determine the feasibility of EV isolation from FWB, we analyzed peripheral venous blood from three donors stored at –80°C for at least one month (Table S2), to simulate the effects of long-term freezing. After thawing, pronounced hemolysis was observed (Figure S1A). Using differential ultracentrifugation (dUC), we obtained EV preparations containing visible reddish precipitates (Figure S1B) and high protein concentrations (Figure S1C). Nanoparticle tracking analysis (NTA) revealed a mean diameter of 134.9 nm (±46.8 nm) and an average particle concentration of 3.5 × 10^11^ (±1.3 × 10^10^) particles/mL (Figure 1C). Transmission electron microscopy (TEM) further confirmed the presence of cup-shaped vesicles with morphology consistent with EVs (Figure 1D). Western blotting was carried out in accordance with minimum information for studies of extracellular vesicles guidelines,^13^ and protein expression was compared between FWB EVs and matched unprocessed FWB samples. CD9 expression was enriched in FWB EV samples compared to the unprocessed FWB (14-fold increase, *p* = 0.001) (Figure 1E). Although CD63 and flotillin-1 displayed ∼5- to 6-fold higher intensity than unprocessed FWB, this did not reach statistical significance (*p* = 0.083 and *p* = 0.113, respectively). Markers of EV-contamination, albumin, ApoA1, ApoE, and calnexin, were present in both unprocessed FWB and EV isolates, with albumin and ApoA1 displaying approximately a 10-fold decrease in signal intensities compared to matched unprocessed FWB samples, although these differences did not reach statistical significance (*p* = 0.22 and *p* = 0.11, respectively). Calnexin and ApoE expressions were significantly decreased in the FWB EV samples (76-fold decrease, *p* = 0.023 and 4-fold decrease, *p* = 0.033, respectively) (Figures 1E and 1F). GAPDH, a cytoplasmic housekeeping protein, was detected at similar levels in both EV isolates and unprocessed FWB (Figures 1E and 1F). Collectively, our findings demonstrate that dUC enables meaningful EV isolation from FWB, and although some contaminants remain detectable, there is relative enrichment of EV markers and depletion of apolipoproteins.

### dUC is a robust, cost-effective and labor-efficient method for isolating EVs from FWB

**Figure 2:**
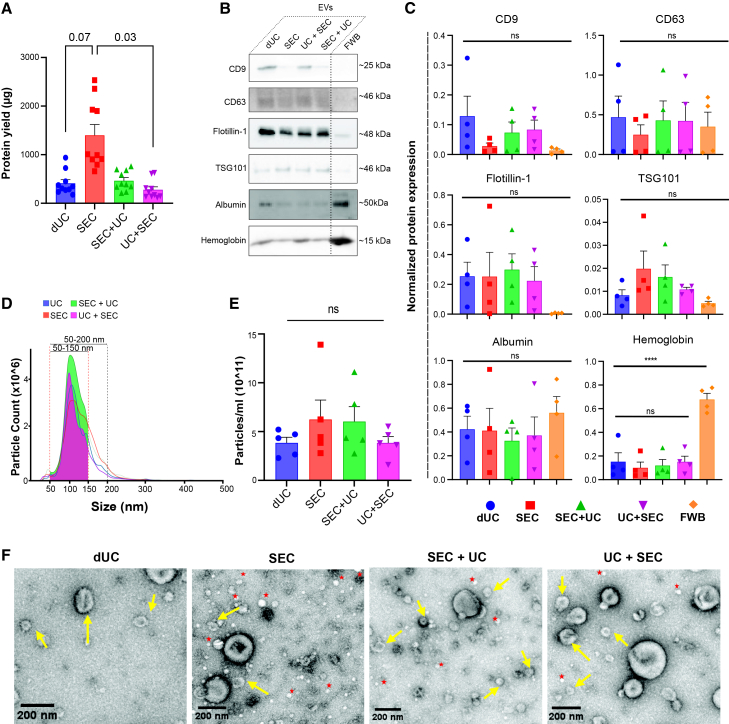
Comparison of EV isolation methods (A) Total yield of protein from FWB EVs isolated from 1 mL FWB using dUC, SEC, SEC + UC, or UC + SEC, assayed by BCA (*n* = 10). (B) Representative immunoblots from FWB EVs isolated by each method from sample X561 and (C) relative protein expression (X483, X533, X561, X577, *n* = 4). (D) Size distribution of particles isolated from FWB by each method (*n* = 5) (X222, X335, X533, X539, X577, *n* = 5). Dotted lines at 50, 150, and 200 nm for common sEV size range. (E) Quantification of particles isolated from 1 mL of FWB by each protocol (*n* = 5). (F) TEM images of particles isolated by each method (X539), yellow arrows denote membrane bound vesicle, red stars denote likely lipoprotein contaminant. Scale bars, 200 nm. ∗∗∗∗*p* < 0.0001, ns, not significant, by one-way ANOVA, with Tukey’s test for multiple comparisons. Error bars represent SEM.

Given that biobanked FWB is often available only in limited volumes, sometimes already aliquoted or partially used, it is particularly important to identify isolation strategies that can maximize EV recovery rate, yield, and purity from such scarce material to enable potential clinical applications. To address this, we systematically evaluated and compared the performance of four isolation protocols (dUC, size-exclusion chromatography (SEC), UC + SEC, and SEC + UC) for the isolation of EVs from 1 mL FWB, a typical aliquot volume used in biobanked samples, as illustrated in Figure 1B. Processing-time varied substantially among protocols, with dUC being the most time-efficient (≈2.5 h), whereas SEC + UC required 3.5 h, SEC alone required 4–7 h, and UC + SEC required 4–8 h (Table S4). Protein quantification of FWB-derived EVs using BCA assay demonstrated that SEC yielded higher protein concentration compared to dUC, UC + SEC, or SEC + UC (Figure 2A), suggesting differences in EV yield and co-isolated protein contaminants.

We further evaluated EV recovery and purity in four FWB-derived EV samples isolated using our four different protocols by western blot (Figures 2B and 2C). dUC, UC + SEC, and SEC + UC displayed stable expression of canonical tetraspanin markers (CD9 and CD63), (Figures 2B and 2C). In contrast, we only detected low levels of CD9 for EVs isolated by SEC alone; however, these samples still displayed expression of TSG101 (Figures 2B and 2C). All methods appeared to enrich flotillin-1 with similar efficacy (Figures 2B and 2C). These results suggest that different isolation methods may differentially recover EV subpopulations. Albumin was detected in EVs isolated by all methods, indicating co-isolation of common plasma components (Figures 2B and 2C). However, Hb, a major component of FWB, was significantly lower in all FWB-derived EV samples compared to unprocessed FWB (*p* < 0.001) and its levels did not differ between the four EV isolation methods (Figures 2B and 2C).

NTA confirmed vesicles predominantly within the 50–150 nm range from all isolation protocols (Figure 2D). The mean size of vesicles was 125.5 ± 41.8 nm for dUC, 133.2 ± 43 nm for SEC, 127.9 ± 42.5 nm for SEC + UC, and 119.3 ± 37 nm for UC + SEC. Vesicles isolated by SEC were larger compared to UC + SEC (*p* = 0.009), while there was no difference observed between other isolation methods (Table S5). Similar ratios of particles/μg were observed across all methods (Table S6). Number of isolated particles for each method was found to be comparable (Figure 2E; Table S7).

TEM further displayed that morphology of FWB-derived EVs were clear cup-shaped vesicles, consistent with EV characteristics, across all four protocols. However, SEC and SEC + UC isolated samples appeared to have higher levels of lipoprotein contamination (Figure 2F), suggesting that initiating the isolation with SEC may enrich lipoprotein contaminants from FWB, whereas dUC and UC + SEC samples exhibited less co-isolates (Table S4).

### EV protein cargo analysis confirms superior performance of dUC and SEC + UC

**Figure 3:**
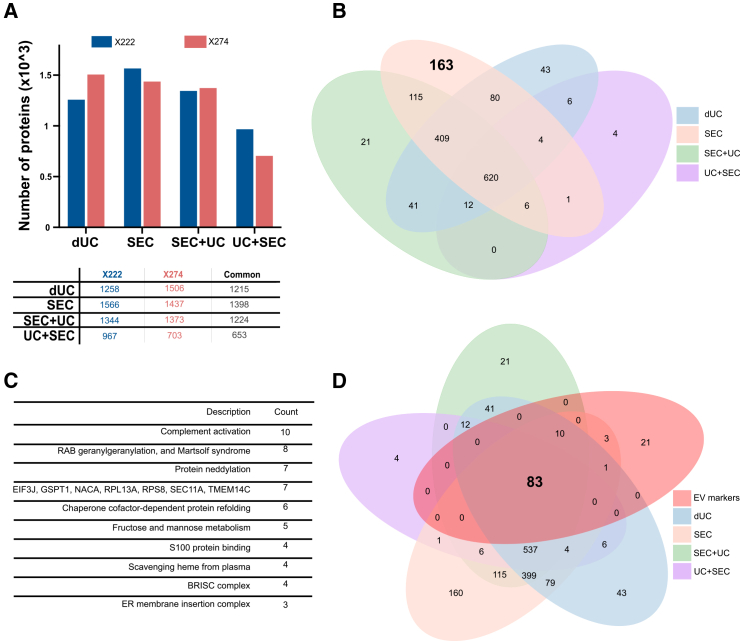
Comparative proteomic profiling of EVs isolated from FWB (A) Total number of detected proteins in EVs isolated from two siNEN samples (X222, X274, *n* = 2) using dUC, SEC, SEC + UC, or UC + SEC. Numbers indicate proteins detected in each sample, with common proteins shared between both samples shown in black. (B) Number of proteins uniquely and commonly identified between methods and present in both X222 and X274. SEC yielded the highest number of unique proteins. (C) STRING clustering of 163 unique proteins detected in SEC (*n* = 2) using a 0.4 interaction score threshold, with the most enrichment in complement factors. (D) Overlap between detected proteins and the top 100 EV markers from Vesiclepedia and Exocarta. All methods shared 83 markers out of 118, whereas only three of the SEC-unique proteins overlapped with top EV markers.

To further evaluate the performance of isolation methods on EV populations obtained, two siNEN samples (X222 and X274) were processed according to all four protocols, and EV cargo was analyzed by mass spectrometry. A total of 1,690 proteins were identified across all methods, defined as proteins detected in at least one sample by any isolation method (Table S8). SEC resulted in the detection of 1,566 and 1,437 proteins in X222 and X274, respectively, with 1,398 proteins shared between both samples (Figure 3A). For dUC, 1,258 and 1,506 proteins were identified, with 1,215 shared proteins. SEC + UC yielded 1,344 and 1,373 proteins, with 1,224 shared between samples. In contrast, UC + SEC resulted in the lowest amount of protein identified, with 967 and 703 proteins detected. While some proteins were uniquely detected in individual samples (Figure S2A; Table S9), these differences are likely to reflect biological variation and may offer insights into inter-individual heterogeneity relevant to personalized medicine. Across methods, dUC, SEC, and SEC + UC showed comparable performance in overall detected proteins and commonly identified across samples, an important factor for biomarker discovery, whereas UC + SEC demonstrated poorer performance.

Comparative analysis revealed that SEC + UC and dUC had similar protein detection profiles, with only 21 and 43 uniquely identified proteins, respectively (Figure 3B) (Table S8). Although SEC yielded 163 unique proteins (Figure 3B), the majority did not map to pathways or functional categories (Figure S2B; Table S10). A small subset of SEC-unique proteins was associated with complement factors and protein folding (Figure 3C), yet only three of those SEC-specific proteins overlapped with a curated list of 118 EV markers (derived from the top 100 reported in Vesiclepedia or ExoCarta). Uniquely identified proteins from other methods did not map to any pathways. In contrast, all methods commonly identified 83 of the 118 curated EV markers (Figure 3D). The comparable performance of isolation methods in proteome coverage, with the exception of UC + SEC, further supports the use of dUC as the most scalable and time-efficient method for EV isolation from FWB and offers robust protein cargo detection.

To further characterize the protein composition of dUC FWB EVs, we compared matched FWB EVs and EV-depleted supernatant from three healthy donors by mass spectrometry. Supernatant fractions showed enrichment of soluble plasma-associated and immune-related proteins, whereas FWB EV showed relative enrichment of cell-associated and signaling-related proteins (Figure S3). These findings indicate that the dUC protocol recovers a protein fraction compositionally distinct from the soluble background of thawed FWB.

### EV isolation from FWB of patients with siNENs enables detection of neuroendocrine-related signature

To assess the potential of our isolation protocol for biomarker discovery, we applied the dUC protocol to a pilot cohort of FWB samples from patients with siNENs and healthy controls. Samples were stratified into two siNEN subgroups, based on the extent of disease at surgical exploration: “advanced” (defined as presence of distant metastasis, i.e., M1 by TNM), and “non-advanced” (defined as M0 by TNM) (Table S1). Mass spectrometry identified on average ∼1,000 proteins per sample (944 ± 40) (Table S11), a protein detection depth within the range reported in plasma EV studies.^14^ In addition, comparison with the same list of 118 unique EV markers derived from Vesiclepedia and ExoCarta revealed an overlap of 91 proteins between FWB siNEN EVs and the curated list. Moreover, there were 346 proteins shared with the plasma EV proteome reported previously from a cohort of 44 healthy controls^15^ (Figure S4).

**Figure 4:**
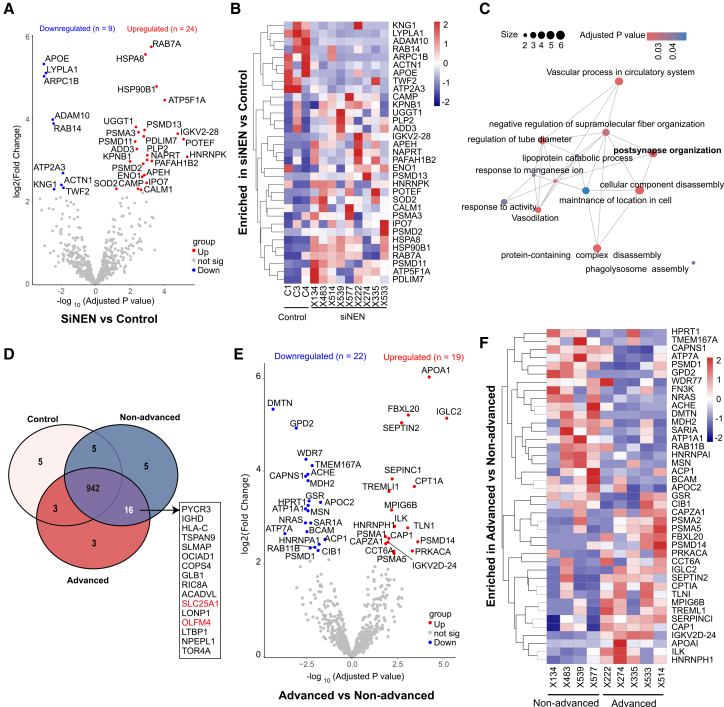
EV proteome reveals synaptic and tumor progression signature in siNEN (A) Volcano plot of differentially expressed proteins in siNEN (*n* = 9) versus control (*n* = 3). Data are plotted as log2(fold change) versus –log10(adj.*p*-value); significantly upregulated proteins (adjusted *p* < 0.1) are highlighted in red, and downregulated proteins in blue. (B) Heatmap of differentially expressed proteins in siNEN-EVs compared to control (scale 2, –2). (C) GO enrichment analysis of siNEN compared to control with redundancy reduction. Node color indicates the adjusted *p* value and node size the number of associated proteins (D) Venn diagram of commonly expressed proteins in at least two samples per group and unique proteins in advanced (*n* = 5) versus non-advanced (*n* = 4). (E) Volcano plot and (F) heatmap of differentially expressed proteins between advanced and non-advanced cases (scale 2,-2).

Mass spectrometry analysis revealed a distinct protein signature in siNEN compared to controls (Figures 4A and 4B; Table S12). Key dysregulated proteins included heat shock protein family A 70 (HSPA8) and heat shock protein 90 beta family member 1 (HSP90B1) (upregulated ∼3-fold, adj. *p* < 0.001 and adj. *p* = 0.002, respectively), POTE ankyrin domain family member F (POTEF) and heterogeneous nuclear ribonucleoprotein K (HNRNPK) (upregulated ∼5-fold, adj. *p* = 0.016 and adj. *p* = 0.031), and ADAM metallopeptidase domain 10 (ADAM10) (downregulated ∼2.5-fold, adj. *p* = 0.007). Pathway-enrichment analysis of differentially expressed proteins revealed significant involvement of synapse organization and neuronal signaling, consistent with the neuroendocrine origin of siNENs (Figure 4C). Notably, 24 proteins were uniquely detected in siNEN-derived EVs, including olfactomedin 4 (OLFM4), previously linked to intestinal inflammation and gastrointestinal malignancies, and solute carrier family 25 member 11 (SLC25A11), associated with neuroendocrine tumorigenesis^16^ (Figure 4D). Comparative analysis between advanced and non-advanced tumors revealed increased expression of apolipoprotein A1 (APOA1) (∼4-fold, adj. *p* < 0.001) and protein kinase cAMP-activated catalytic subunit alpha (PRKACA) (∼3-fold, adj. *p* = 0.088) in advanced siNENs, reflecting alterations in lipid-metabolism and liver-associated pathways, a decrease of acetylcholinesterase (ACHE) (∼2-fold, adj. *p* = 0.008), as well as increased heterogeneous nuclear ribonucleoprotein H1 (HNRNPH1) (∼2-fold, adj. *p* = 0.043), a regulator of neuron differentiation (Figures 4E and 4F; Table S13).

Together, these findings demonstrate that EVs isolated from biobanked FWB capture tumor- and phenotype-specific molecular signatures, supporting their feasibility for retrospective biomarker discovery in rare malignancies such as siNEN.

## Discussion

The suitability of biobanked FWB samples for EV analysis remains largely unexplored. FWB has been considered an unsuitable source for proteomics applications due to extensive RBC lysis, protein aggregation, and variable pre-analytical handling,^17^^,^^18^ restricting its use mainly to DNA or RNA extraction.^9^ However, EV-enclosed membrane structure may retain their molecular integrity even after long-term storage. Thus, while FWB is suboptimal for bulk proteomics, it could still provide a viable source of EVs. Here, we demonstrate that even small volumes of FWB (1 mL), stored from months to years, can yield intact EVs suitable for downstream proteomic analysis. Our findings challenge the conventional view that FWB is unsuitable for proteomic studies and demonstrate that EVs isolated from FWB provide a biologically informative proteome (e.g., captured neuroendocrine-related molecular signatures in FWB-derived EVs samples from siNEN patients) with reduced blood contaminants. Building on these findings, we established a robust and reproducible dUC-based workflow with quality control by NTA, TEM and western blotting, enabling reliable downstream proteomics and capturing tumor-specific molecular features.

Using FWB from healthy donors and siNEN patients, we compared dUC, SEC, and combined (UC + SEC or SEC + UC) isolation methods. Our results showed that dUC performed comparably to SEC and combined methods, while requiring the least processing time. This contrasts with the findings by Wan et al., who reported that a modified dUC protocol, with either addition of washing step (UC3), or SEC following dUC (UC2 + SEC), significantly enhanced EV purity from plasma.^14^ The discrepancy likely reflects differences in sample type and experimental context. The protocols of Wan et al. were optimized for large-volume (e.g., 8 mL for UC3 and up to 40 mL for UC2-qEV) fresh plasma, which contains low levels of blood cell-derived proteins, whereas our study focused on low-volume (∼1 mL) of FWB that is rich in Hb released from lysed RBC.^17^^,^^18^ In plasma samples with mild Hb contamination, SEC theoretically removes monomeric albumin that is eluted in later fractions while tending to co-elute Hb aggregates with EVs in early fractions.^19^^,^^20^ However, in FWB samples, this behavior becomes unpredictable because freeze-thaw cycles of FWB release high abundance of Hb, together with platelet- and leukocyte-derived proteins and microparticles, which have high potential to form mixed aggregates that may alter column flow and impair protein separation efficiency.^10^^,^^19^ Consistent with this, in our study, SEC yielded the highest total protein recovery but did not significantly reduce Hb or albumin compared to other isolation methods, and TEM suggested increased lipoprotein co-isolation. These observations indicate that the compositional and particulate complexity of FWB compromises the performance of SEC, limiting its purification advantage despite higher protein yield. In contrast, dUC is less affected by such aggregation-prone conditions and therefore provides the best balance between yield, purity, and scalability for FWB samples among the tested methods (Table S4).

Beyond method performance, we next considered whether the protein cargo detected in dUC-isolated FWB EVs was dominated by co-isolated soluble proteins from the post-thaw background. Our side-by-side comparison with matched EV-depleted supernatants confirmed that dUC-isolated FWB EVs showed distinct proteomic signature enriched in cell-associated, membrane-associated, and signaling-related proteins, whereas the supernatants were enriched for soluble plasma- and immune-related proteins. Proteins of FWB EV also overlapped with 91 of 118 curated EV-associated proteins from Vesiclepedia and ExoCarta (77.1%) and shared 346 proteins with published plasma EV proteome reported by Hoshino and colleagues.^15^ These findings support that FWB EVs retain recognizable EV-associated protein cargo rather than simply reflecting the soluble protein background.

However, these overlaps should not be interpreted as evidence that FWB EVs are equivalent to plasma-derived EVs. Unlike plasma, FWB undergoes freeze-thaw associated cellular disruption and hemolysis, which may affect both EV recovery and composition. Some vesicles may be lost with Hb- or other protein-associated aggregates during debris removal, whereas freeze-thaw-associated stress and damage to RBCs, leukocytes, and platelets may contribute additional blood cell-derived vesicles.^21^^,^^22^ Thus, FWB EVs should be viewed as a matrix-specific composite EV pool, potentially containing pre-existing circulating EVs, blood cell-derived vesicles released during freeze-thaw, and co-isolated background components. Although such effects are often regarded as technical noise in conventional plasma EV studies, in biobanked FWB, they may represent part of a composite molecular profile influenced by blood cell stress, storage response, and disease-associated systemic changes. Previous studies suggest that both disease state and storage-related stress can influence blood-cell-derived EV release, raising the possibility that storage-associated vesicle release may vary between individuals rather than representing only uniform technical noise.^10^^,^^23^ In cancer, where inflammation, immune dysregulation, and hypercoagulability coexist, such composite EV profiles could offer valuable insights into systemic disease alterations rather than simple experimental artifacts.^24^ However, as only biobanked FWB siNEN samples were available, we could not distinguish the relative contributions of pre-existing circulating and freeze-thaw induced EVs, nor could we determine the biological relevance of such storage-associated components. This question will require dedicated investigation in future studies using paired fresh and FWB samples, disease-matched controls, and cell-type-specific or single-vesicle analyses.

Importantly, our findings highlight the feasibility of using biobanked FWB for retrospective biomarker discovery, especially in rare diseases where prospective sampling is difficult. We validated this in a small siNEN cohort, a rare malignancy with an incidence of ∼1 per 100,000.^12^ Despite the small cohort and lack of a siNEN reference proteome, EV isolated from FWB revealed enrichment of postsynaptic organization pathway and differences between advanced and non-advanced disease. Dysregulated factors between siNEN groups included acetylcholinesterase and hnRNPs, suggesting a disease-associated EV profile. OLFM4, uniquely detected in siNEN samples, has been shown elevated in certain gastrointestinal cancers such as pancreatic and colorectal cancers, supporting the biological relevance of our findings.^15^^,^^25^ This encourage the use of FWB-derived EVs for other rare diseases, for example application to national initiatives such as the Swedish Childhood Tumor Biobank, which has been collecting over 1,500 whole blood samples since 2013, or international biorepositories, such as Reprocell, encompassing samples from over 120,000 donors.^26^

Beyond proteomics, the approach could be extended to other EV cargo. EV-RNA cargo has shown great promise as biomarker in studies of plasma,^27^^,^^28^ consistent with a recent study identifying a 51 gene RNA signature from FWB outperforming serum chromogranin A for NEN diagnosis.^29^^,^^30^ Likewise, EV-metabolomics of plasma-derived EVs has revealed glycerophospholipid dysregulation associated with gastric cancer.^31^ Transcriptomic and metabolomic analysis of FWB-derived EVs may therefore enhance retrospective molecular profiling in rare cancers.

In summary, we developed a dUC-based workflow that reproducibly recovers EV-enriched fractions from small-volume archived FWB, yielding material suitable for downstream protein-cargo analysis. Pilot data from siNEN samples shows that FWB EVs can capture disease-associated proteomic features, supporting the use of biobanked FWB, beyond conventional nucleic acid-based analyses, for retrospective EV-oriented biomarker studies, particularly in rare diseases, where prospective sample collection is challenging. Future validation in larger, independent cohorts is warranted to establish the clinical biomarker value of FWB EVs.

### Limitations of the study

Some unique proteins detected by each method may be of biological interest, and in specific cases, isolation methods could be optimized for target applications. This study focused on feasibility and protocol benchmarking rather than biomarker discovery, and the small cohort limits the statistical power. The study asked whether 1 mL FWB starting volume was sufficient to isolate EVs from, but the low volume may limit the coverage of EV subpopulations. The absence of reference proteomes for siNEN, and potential differences between FWB, plasma EVs and native circulating EVs, requires validation. Finally, as this study focused on protein cargo for the confirmation of EV isolation and evaluating utility of FWB, profiling of other cargo in FWB-derived EVs remains to be explored.

## Resource availability

### Lead contact

Requests for further information and resources should be directed to and will be fulfilled by the lead contact, Linda Bojmar (linda.bojmar@liu.se).

### Materials availability

This study did not generate new unique reagents.

### Data and code availability

• The mass spectrometry proteomics data have been deposited to the ProteomeXchange Consortium via the PRIDE^32^ partner repository with the public dataset identifier PXD072143.
• This study does not report original code.
• Any additional information required to reanalyze the data reported in this study is available from the lead contact upon request.

## Acknowledgments

The authors extend our gratitude to members of the Bojmar laboratory for insightful comments. We also extend our deep gratitude to Professor Anna Fahlgren for help with dissemination of the work. We thank the 10.13039/501100004359Swedish Research Council (2021-02356), the 10.13039/501100002794Swedish Cancer Society (211824 Pj, 243887 Pj, and 254208 IA), 10.13039/501100003748Swedish Society for Medical Research (S21-0079) and Region Östergötland research-ALF (RÖ-1023413) for funding. We acknowledge the Core Facility at the Faculty of Medicine and Health Sciences, Linköping University for providing assistance in mass spectrometry and electron microscopy. The authors thank the LiU-EV network for providing instrumentation and expertise for this research.

## Author contributions

Conceptualization, C.F. and L.B.; methodology, J.B., C.F., and L.B.; investigation, J.B., C.F., P.V.R., and A.S.; formal analysis, J.B., C.F., M.E.H., and P.V.R.; writing – original draft, J.B., C.F., M.E.H., and L.B.; writing – review and editing, J.B., C.F., M.E.H., P.V.R., A.S., R.K., L.A., C.P.Z., O.G., and L.B.; funding acquisition, L.B.; resources, M.E.H.; supervision, O.G. and L.B.

## Declaration of interests

The authors declare no conflicts of interest.

## Declaration of generative AI and AI-assisted technologies in the writing process

During the preparation of this work, the authors used ChatGPT 5.0 in order to refine English grammar of the abstract. After using this tool or service, the authors reviewed and edited the content as needed and take full responsibility for the content of the publication.

## STAR★Methods

### Key resources table

**Table:** 

REAGENT or RESOURCE	SOURCE	IDENTIFIER
**Antibodies**		

Anti-Calnexin Mouse Antibody (AF18)	Thermo Fisher Scientific	Cat# MA3-027; RRID: AB_2069043
Anti-Hemoglobin Polyclonal Antibody	Thermo Fisher Scientific	Cat# PA5-145254; RRID: AB_3094049
Anti-CD9 Recombinant Rabbit Monoclonal Antibody (SA35-08)	Thermo Fisher Scientific	Cat# MA5-31980; RRID: AB_2809274
Anti-Human CD63 antibody (H5C6)	BD Biosciences	Cat# 556019; RRID: AB_396297
Mouse Anti-Flotillin-1 (18)	BD Biosciences	Cat# 610821; RRID: AB_398139
Mouse Anti-Human Albumin antibody BGN/1328/33 (UA33)	Bio-Rad	Cat# 0300-0080; RRID: AB_617171
Anti-Human ApoA1 Mouse antibody (5F4)	Cell Signaling Technology	Cat# 3350; RRID: AB_2227261
ApoE (pan) (D7I9N) Rabbit Monoclonal Antibody	Cell Signaling Technology	Cat# 13366; RRID: AB_2798191
Anti-TSG101 antibody [EPR7130(B)]	Abcam	Cat# ab125011; RRID: AB_10974262
GAPDH Antibody (0411)	Santa-Cruz Biotechnology	Cat# sc-47724; RRID: AB_62767
Goat Anti-Mouse Antibody	Agilent	Cat# P0447; RRID: AB_2617137
Goat Anti-Rabbit Antibody	Agilent	Cat# P0488; RRID: AB_3718618

**Biological samples**		

Human frozen blood samples	Linköping University Hospital	This paper

**Critical commercial assays**		

Pierce™ BCA Protein Assay	Thermo Fisher Scientific	Cat# 23225

**Deposited data**		

Raw proteomic data	This paper	PXD072143

**Software and algorithms**		

Graphpad Prism 10.4.2	Graphpad	RRID: SCR_002798
R Project for Statistical Computing 4.4	R Core Team	RRID:SCR_001905
Spectronaut v.19	Biognosys	N/A
RADIUS EM Imaging Software v.2	EMSIS GmbH	N/A
NTA v.3.4	Nanosight	RRID:SCR_014239
ImageJ2	Fiji	RRID:SCR_002285

**Other**		

Automatic Fraction Collector V2	IZON Science	AFC-V2
qEV2 column	IZON Science	qEV2 gen 2
Nanoparticle tracking Nanosight 300	Malvern Panalytical	NS300; RRID: SCR_027852
Ultracentrifuge XPN-90	Beckman Coulter	RRID: SCR_018238
Rotor type 50.4TI	Beckman Coulter	Cat# 347299

### Experimental model and study participant details

#### Human samples

Blood from patients with siNENs undergoing resections between 2012-2021 was collected perioperatively and banked for future analysis. The study was approved by the Regional Ethics Committee in Linköping (Dnr 2010/214-31). The participants’ written informed consent was obtained prior to inclusion. A subset of 10 patients was selected for the purpose of this study. Patients’ characteristics are summarized in Table S1.

### Method details

#### Literature search

A literature search was conducted on July 27th, 2025, through PubMed. The search strategy used to identify all potential studies of circulating extracellular vesicles using FWB in humans as follows: (((((extracellular vesicles[Title/Abstract]) OR (exosom∗[Title/Abstract])) OR (microvesicles[Title/Abstract])) OR (microparticles[Title/Abstract])) OR (circulating microparticles[Title/Abstract])) AND ((((((((((((cryopreserved whole blood[Title/Abstract]) OR (Cryopreservation of Whole Blood[Title/Abstract])) OR (Whole Blood Cryopreservation[Title/Abstract])) OR (frozen whole blood[Title/Abstract])) OR (blood preservation[Title/Abstract])) OR (blood storage[Title/Abstract])) OR (biobanking[Title/Abstract])) OR (cryopreserv∗[Title/Abstract])) OR (cryopreserved[Title/Abstract])) OR (cryopreservation[Title/Abstract])) OR (blood preservation[MeSH Terms])) OR (blood preservations[MeSH Terms])).

#### Sample preservation

Blood from siNEN patients was obtained perioperatively in EDTA tubes, and was stored at −80°C before further processing. Six milliliters of blood from healthy donors were collected in EDTA tubes, and stored at -80°C for at least a month before further processing. Sample preservation conditions are summarized in Table S2.

#### EV isolation

FWB samples stored at −80°C were thawed at room temperature for 20 minutes before being divided into 4 different groups for EV isolation to compare implications of isolation methods from 1 mL FWB. EDTA tubes with samples were only thawed once to reduce the impact of repeated freeze-thaw cycles. All samples were preprocessed by centrifugation at 500 × g for 10 minutes (10°C), and the supernatant was transferred for another centrifugation step at 3000 × g for 20 minutes (10°C) to pellet cellular debris and platelets. The supernatant was subsequently transferred to a new tube without disturbing the pellet, and centrifuged at 12 000 × g for 20 minutes (10°C) to further remove debris and microvesicles from the sample. dUC for EV isolation was performed as previously described,^33^ briefly, following preprocessing, the supernatant was transferred to ultracentrifuge tubes and subjected to 100 000 × g for 70 minutes at 10°C on an XPN-90 ultracentrifuge using a 50.4TI rotor (Beckman Coulter, Indianapolis, IN) to pellet EVs.The pellet was then washed in 3 mL ice-cold PBS before being centrifuged at 100 000 × g for 70 minutes at 10°C using a 50.4TI rotor, finally resuspending the pellet in 100 μL PBS for downstream analysis. For exploratory comparison with matched FWB EVs, EV-depleted supernatant was collected from a subset of samples as indicated. Briefly, the supernatant obtained after the first 100 000 × g pelleting step was subjected to a second 100 000 × g for 70 minutes at 10°C to further deplete residual vesicles using same rotor as FWB EVs preparation. The resulting supernatant was collected as the EV-depleted supernatant.

Size exclusion chromatography (SEC) was conducted using qEV2 (Izon, Christchurch, New Zealand) according to the manufacturer’s protocol. Briefly, the column was washed using 90 mL PBS, 1 mL FWB was mixed in equal part PBS, and the mixture was loaded onto the frit following preprocessing as described above. The 15.5 mL void volume was discarded, and 4 fractions of 2 mL were collected. These 4 fractions were subsequently pooled and concentrated using 15 mL MWCO 100 kDa Centrifugal Filter Units (Amicon, Merck Millipore, Burlington, MA) down to 100-200 μL by centrifugation at 3900 × g, rotating the filter unit 180 degrees every 20 minutes. In the UC + SEC group, following preprocessing, samples were subjected to 100 000 × g for 70 minutes at 10°C on an XPN-90 ultracentrifuge using a 50.4TI rotor. The pellet was then further purified by resuspending in 2 mL PBS, and ran on qEV2 (Izon) as described above. The SEC + UC was conducted in the same manner as the SEC alone, but instead of concentration method from using 100 kDa Centrifugal Filter Units, the column effluent was subjected to UC at 100 000 × g for 70 minutes at 10°C, as above, and finally resuspending the pellet in 100 μL PBS for downstream analysis.

#### EV characterization

EV protein was quantified by BCA Protein Assay (Pierce, ThermoFisher Scientific, Waltham, MA) by incubating at 37°C for 30 minutes and read at 562 nm on Versamax Microplate reader (Molecular Devices, San Jose, CA) in technical duplicates. Unprocessed FWB was diluted 1:10 for comparison. Following protein quantification, samples were aliquoted in 5 and 10 μg aliquots for downstream analysis to reduce the number of freeze-thaw cycles of EV samples. Size distribution was determined by nanoparticle tracking on NS300 (Malvern Panalytical, Malvern, United Kingdom) as previously described,^33^ briefly samples were diluted 1:1000 in PBS and injected into the flow cell via a syringe pump (Malvern Panalytical) at speed 60 (A.U). Five videos of 30 seconds were captured using the 488 nm laser at camera level 12, and particles were tracked and analyzed at detection threshold 5 using Nanosight NTA software (v.3.4, Malvern Panalytical).

EV size and morphology were further validated by negative staining. Briefly, a formvar carbon-coated copper EM grid was placed on a 5 μL drop of EV sample, and incubated for 10 minutes at RT. Excess sample was removed by blotting the grid with filter paper and washed twice using 20 μL of deionized water. Following washing steps, negative staining was performed by placing the grid on a drop of 2% uranyl acetate for 30 s, blotted again with filter paper, and air-dried in an EM grid box. Electron micrographs were acquired using a JEOL JEM-1400 Flash transmission electron microscope (JEOL Ltd., Tokyo, Japan) operated at 80 kV and equipped with a XAROSA camera and RADIUS software (EMISIS GmbH, Münster, Germany).

#### Western blot

EVs isolated by each method were lysed using RIPA buffer and Laemmli SDS buffer supplemented with 2-mercaptoethanol. 4 or 8 μg EV protein were heat-denatured at 95°C for 5 minutes, and separated on a 4-20% gel by SDS-PAGE (Bio-Rad, Hercules, CA) at 100 V for 30 minutes, followed by 150 V for 35 minutes. Proteins were then transferred to a PVDF membrane using a Trans-blot Turbo cassette (Bio-Rad, Hercules, CA) and the TGX-mini protocol, 2.5 A, 25 V for 3 minutes.

Membranes were blocked for 60 minutes in 5% BSA in TBS (Bio-Rad), and stained overnight at 4 °C in primary antibody against CD9 (1:1000, SA35-08, Thermo Fisher Scientific), CD63 (1:1000, H5C6, BD Biosciences, Franklin Lakes, NJ), TSG101 (1:1000, EPR7130(B), Abcam, Cambridge, UK), Flotillin-1 (1:1000, Clone 18, BD Biosciences), GAPDH (1:2000, sc-47724, Santa Cruz Biotechnology, Dallas, TX), calnexin (1:1000, AF18, Thermo Fisher Scientific), ApoA1 (1:1000, 5F4, Cell Signaling Technology), ApoE (1:1000, D7I9N, Cell Signaling Technology), albumin (1:2000, BGN/1328/33 (UA33), Bio-Rad), and hemoglobin (1:2000, PA5-145254, Thermo Fisher Scientific). Membranes were washed in TBS-T (0.1% Tween), and incubated with HRP-conjugated goat secondary antibody, raised against the host species of the primary antibody (1:5000 Dako, Agilent Technologies, Santa Clara, CA) for 60 minutes at room temperature. Signal was detected on a ChemiDoc Imaging System using Clarity Max™ Western ECL Substrate (Bio-Rad) and bands quantified using Image Lab (v.6.1.0 build 7, Bio-Rad), with ApoE homodimers being quantified as previously described.^34^ For method comparison, two gels were ran featuring samples from two patients per gel, for each of the four isolation methods, which were compared to unprocessed FWB. Band intensities were normalized against total protein loaded by Stain-Free quantification.

#### Mass spectrometry

EVs were lysed using RIPA and sonication for 10 minutes, followed by reduction using 25 μL 8 M Urea in 50 mM ammonium bicarbonate, and 10 mM DTT and placed on a shaker at 900 RPM for 1 h. Following reduction, samples were alkylated by adding 5 μL iodoacetamide and placed back on the shaker for 1 h at 37°C while protected from light. Following alkylation, urea was diluted and 0.1 μg trypsin in 50 mM ammonium bicarbonate was added, and samples were digested overnight at 37°C after which the reaction was stopped by acidifying the solution to pH 2-3 using trifluoroacetic acid. Samples were desalted and cleaned using C18 filter tips (Pierce, Thermo Fisher Scientific) and eluted using 50% acetonitrile in 0.1% formic acid. QC and quantification were carried out by Nanodrop (Thermo Fisher Scientific) to detect peptide concentration by absorbance at 280 nm. Aliquoted peptide samples were dehydrated by Speedvac (Thermo Fisher Scientific) and stored at -80°C before analysis.

LC-MS was carried out on a Bruker TIMS-TOF Pro mass spectrometer coupled with a Pepsep Xtreme, 25 cm, 150 μm inner diameter, 1.5 μm particle size (Bruker Daltonics) and operated in data-independent mode. Briefly, 600 ng sample was loaded at a rate of 400 nL/min on a 45 min gradient of 0.1% formic acid in water (A) and 0.1% formic acid in acetonitrile (B) as follows: from 2% B to 17% B over 25 minutes; from 25% B to 37% B over 35 minutes; from 37 % B to 95% B until 45 minutes. Column temperature was kept at 50°C. Peptides were ionized in a Captive nano-electrosprayer (Bruker) before being injected into the mass spectrometer. Data was acquired in DIA-PASEF mode with a MS1 scan range of 100-1700 *m/z*, 21 isolation windows of 25 Da each were tiled continuously from 475 to 1000 Da, and collision energy was linearly interpolated between 1/K0 values, from 20 eV at 0.6 Vs/cm2 to 59 eV at 1.6 Vs/cm2, keeping constant above or below.

Raw data was deposited to PRIDE (PXD072143) and proteins were identified against human reference proteome UniProt (UP000005640) using Spectronaut (v.19.4 and v.19.9, Biognosys, Zurich, Switzerland) in directDIA+ (Deep) mode using the following settings: digestion using Trypsin/P with a maximum of 2 missed cleavages was used as cleavage rules. The minimum and maximum peptide lengths were set to 5 and 52 amino acids, respectively. Carbamidomethylation of cysteine was set as a fixed modification, while acetylation of protein N-terminus and oxidation of methionine were included as variable modifications, with a maximum of 5 variable modifications per peptide. Quantity was based on MS2 AUC. The FDR threshold for protein, peptide and PSM identification was set to 0.01.

Low intensity proteins were excluded based on density distribution, and proteins with missing values were filtered prior to differential analysis. The remaining intensities were quantile normalized using the *normalizeBetweenArrays()* function from the limma_3.62.2 package. Differential expression analysis was performed with robust linear modeling and empirical Bayes moderation. Proteins with |log_2_FC| > 1 and FDR-adjusted *p*-value <0.1 were considered significant. Gene Ontology enrichment (biological processes) of significant proteins was performed using *enrichGO()* from clusterProfiler_4.14.6 package. Proteins filtered were further evaluated for group-specific expression patterns, and Venn diagrams were constructed to compare protein presence across groups. A curated list of 118 EV markers was generated by combining the unique proteins from the top 100 most frequently reported proteins of both Exocarta^35^ and Vesiclepedia.^36^

### Quantification and statistical analysis

Data is presented as mean ± standard error of the mean (SEM) unless otherwise stated. Individual data points in bar plots represent independent biological replicates (n) from different donors. Group comparisons were conducted by Student’s t-test, or one-way ANOVA followed by Tukey’s test for post-hoc analysis of multiple groups. Statistical analysis was conducted using GraphPad Prism v.10.4.2 for Windows (GraphPad Software, Boston, MA). *p* < 0.05 was deemed statistically significant. Additional statistical details are described in the corresponding figure legends. Protein-protein interactions were analyzed using STRING-DB (v.12.0) using an interaction score cut-off of 0.4.

## Supplemental information

Document S1. Figures S1–S4 and Tables S1, S2, S4–S7, S12, and S13

Table S3. Summary of search results, and reason for exclusion, related to Figure 1

Table S8. Proteins detected by mass spectrometry by each method (*n* = 2)

Table S9. Unique proteins from each patient detected by mass spectrometry (*n* = 2), related to Figure 3

Table S10. Protein-protein interaction scores for proteins uniquely present in EVs isolated by SEC using STRING DB, related to Figure 3C

Table S11. Proteins detected by mass spectrometry, related to Figure 4

Data S1. Raw western blot images corresponding to Figures 1 and 2
