# Supplementary material for: Extracellular vesicles isolated from frozen whole blood show biomarker potential in small intestine neuroendocrine neoplasms
Source: iScience. 2026 Jul 14;29(8):116723. doi: 10.1016/j.isci.2026.116723 (PMC13382587; doi:10.1016/j.isci.2026.116723)
Supplement: Document S1. Figures S1–S4 and Tables S1, S2, S4–S7, S12, and S13 [file mmc1.pdf]

iScience, Volume *29*

Supplemental information

Extracellular vesicles isolated from frozen whole blood show biomarker potential in small intestine neuroendocrine neoplasms

Jonas Burman, Chuanwen Fan, Mohamed El Husseiny, Paulina Velasco Riestra, Alexander Sandberg, Constantinos P. Zambirinis, Róbert Kotán, Lúcia Amorim, Oliver Gimm, and Linda Bojmar

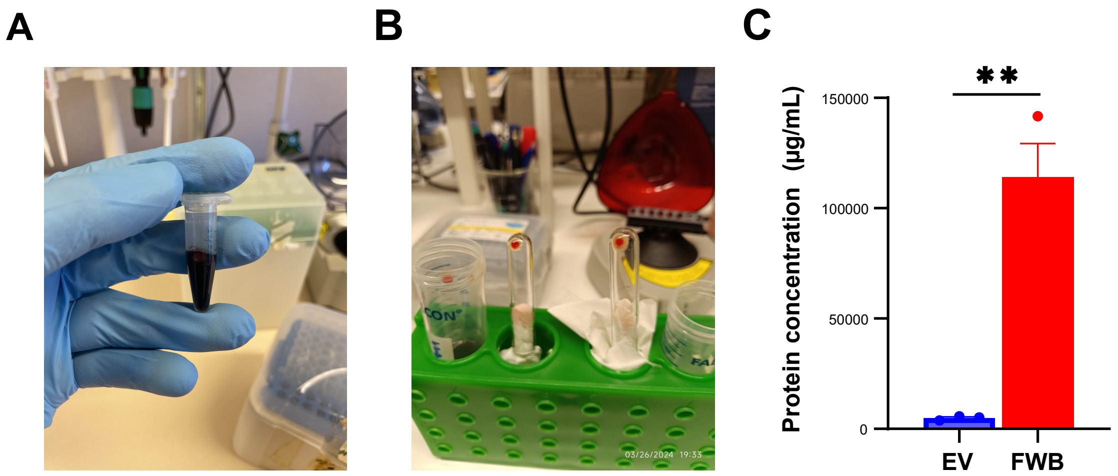

Figure S1: FWB samples show high degree of hemolysis A) FWB sample following centrifugation at 12 000 x g displaying hemolysis. B) FWB samples following dUC displaying clear red pellet. C) Protein concentration of FWB EV samples compared to unprocessed FWB samples quantified by BCA (n = 3). ** $p < 0.01$ by Student's T-test, error bar represents SEM

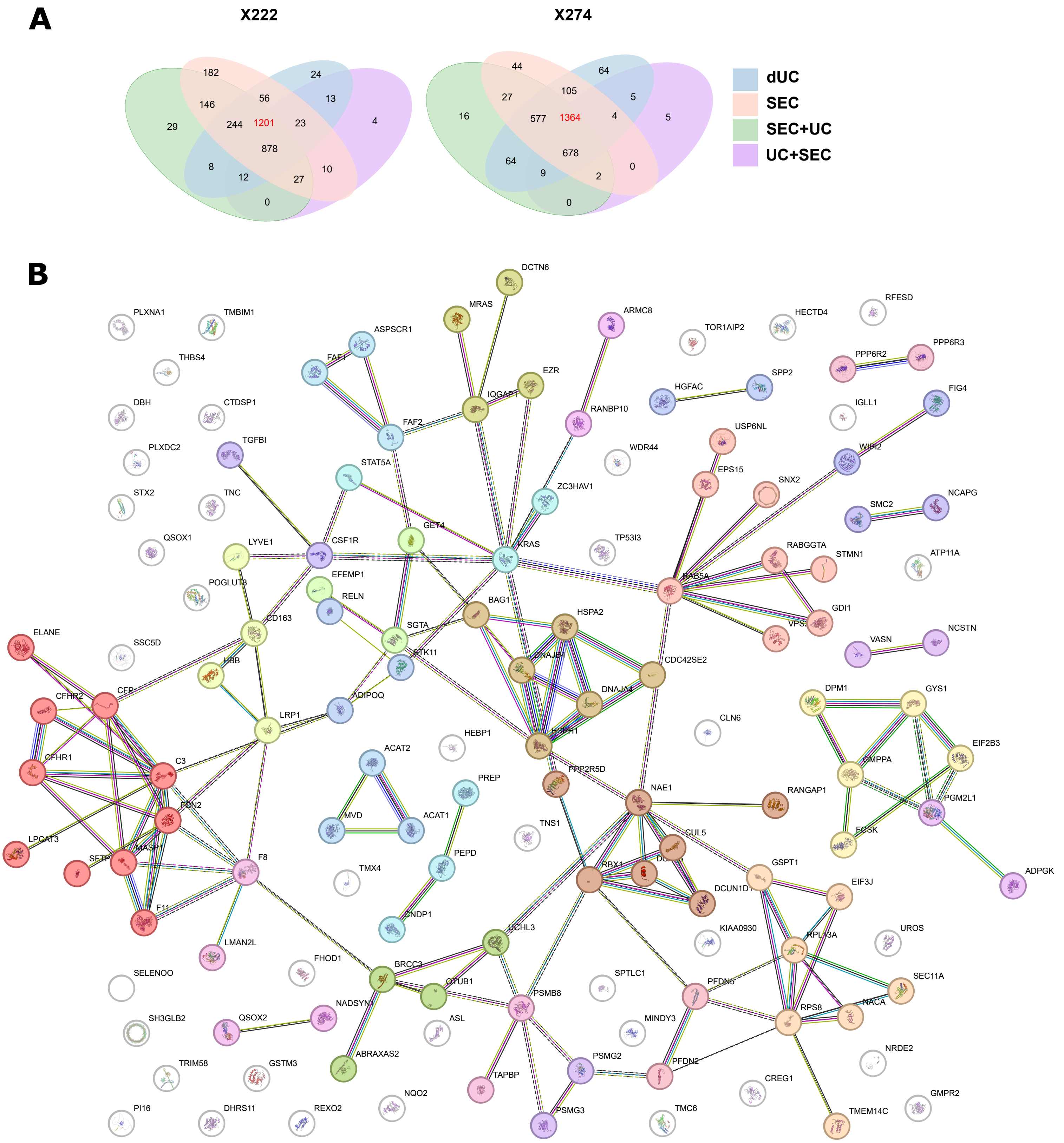

Color	Description
	Complement activation
	RAB gernylgernylation, and Martsolf syndrome
	Protein neddylation
	EIF3J, GSPT1, NACA, RPL13A, RPS8, SEC11A, TMEM14C
	Chaperone cofactor-dependent protein refolding
	Fructose and mannose metabolism
	S100 protein binding
	Scavanging heme from plasma
	BRISC complex
	ER membrane insertion complex
	Signaling by FLT3 ITD and TKD mutants
	+Dipeptidase activity
	+UAS
	Terpenoid backbone biosynthesis

Color	Description
	AMPK inhibits chREBP transcriptional activity
	HGFAC, SPP2
	FIG4, WIPI2
	+Positive regulation of chromosome condensation
	CSF1R, TGFBI
	Proteasome assembly
	NCSTNm VASN
	ADPGK, PGM2L1
	CTLH, C-terminal LisH motif, and GID complex
	NADSYN1, QSOX2
	Cargo concentration of the ER
	PSMB8 TAPBP
	PPP6R2, PPP6R3
	Prefoldin comple

Figure S2. Method specific protein identification. A) Total proteins identified by each EV isolation method in X222 and X274. B) Mapping of unique proteins from SEC to clusters using STRING. Related to Figure 3.

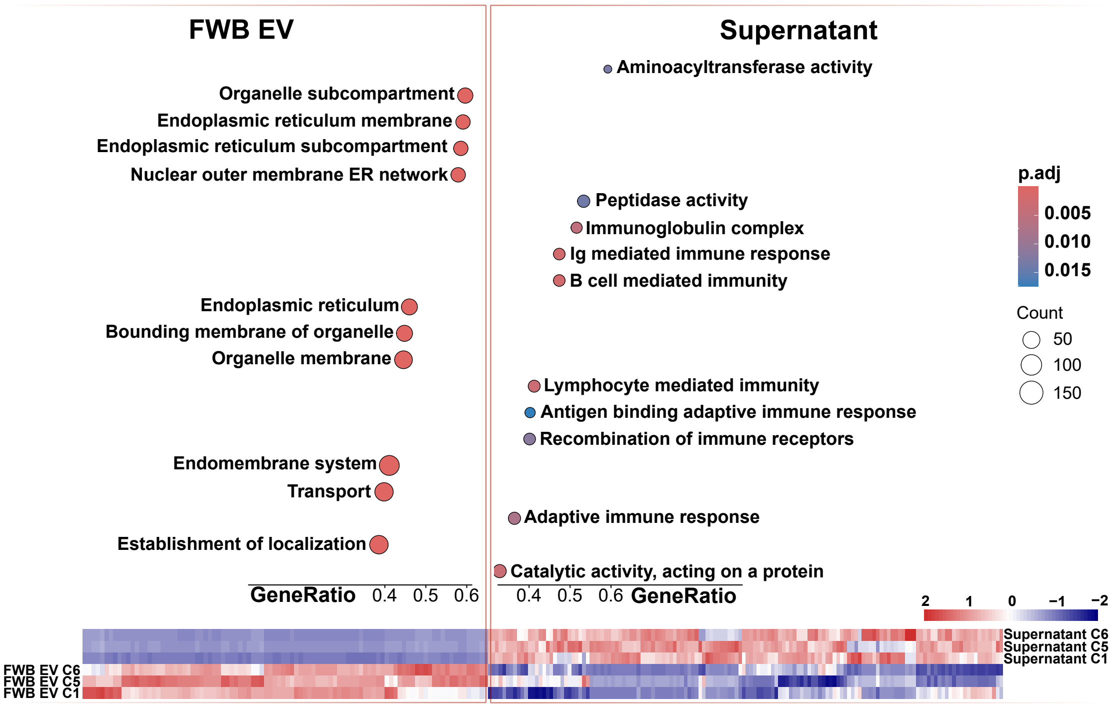

Figure S3. Comparison of FWB EV and matched FWB supernatant proteomes from three healthy controls. Supernatant fractions showed enrichment of soluble plasma-associated proteins and immune-related pathways, whereas FWB EVs showed relative enrichment of cell-associated and signaling-related proteins. n = 3

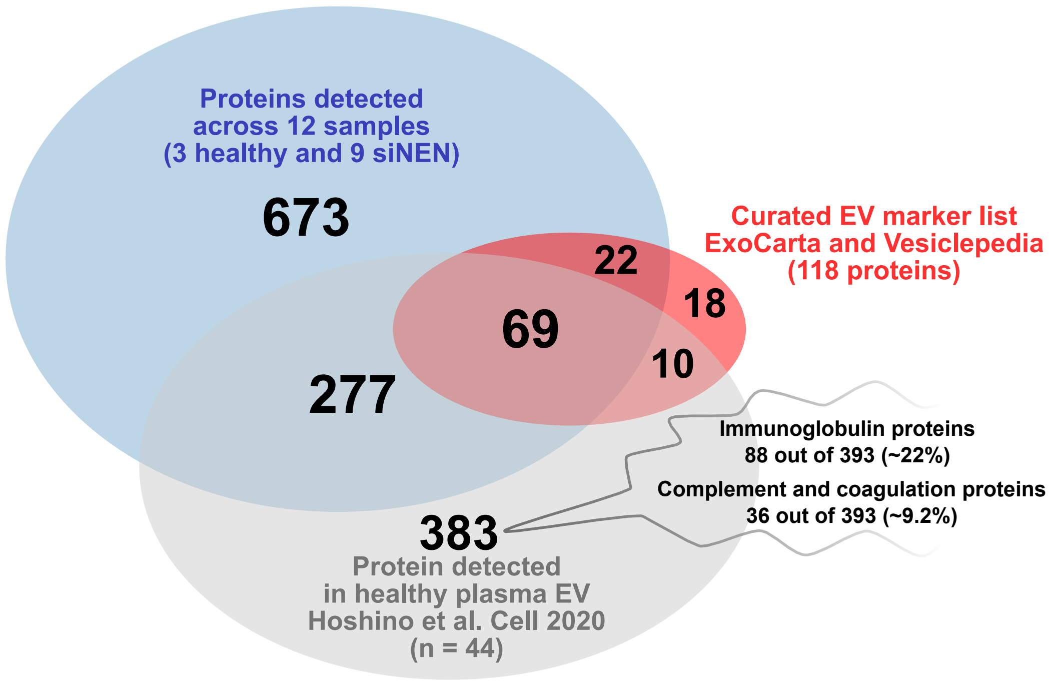

Figure S4. Overlap of FWB EV proteome with curated EV marker list, and plasma EV proteins reported by Hoshino et al. Cell 2020. FWB EVs shared a large overlap with known EV-associated proteins (91 out of 118). Plasma EV unique proteins were enriched in soluble plasma-associated proteins.

Table S1. Summary of patient characteristics. Median age and interquartile range for patients included. TNM category for patients included reported in pT, pN and M

Characteristics	N = 10, n(%), or median (IQR)
**Age**	64 (IQR 58-73.75)
**Male sex**	6 (60%)
**Categories**	
**T1**	1
**T2**	1
**T3**	2
**T4**	6
**N0**	2
**N1**	4
**N2**	4
**M0**	4
**M1**	6

Table S2. FWB sample storage information. Related to Figure 4

Sample ID	Collection date	Group	Collection tube	Freeze-thaw cycles	Identified proteins
C1	2/5/2024	Healthy	EDTA	1	988
C3	3/10/2025	Healthy	EDTA	1	896
C4	4/14/2025	Healthy	EDTA	1	978
134	7/4/2012	Non-advanced	EDTA	1	989
222	1/13/2014	Advanced	EDTA	1	949
274	8/28/2015	Advanced	EDTA	1	975
335	6/11/2015	Advanced	EDTA	1	896
483	9/5/2018	Non-advanced	EDTA	1	895
514	8/29/2019	Advanced	EDTA	1	887
533	1/9/2020	Advanced	EDTA	1	969
539	2/20/2020	Non-advanced	EDTA	1	962
577	8/18/2021	Non-advanced	EDTA	1	971

Table S4. Summary of isolation methods. Estimated time for the isolation of EVs from 1 mL FWB in hours, variance in time due to sample concentration using ultrafiltration following SEC. Observed purity by reduction of co-isolates based on TEM and protein quantity. Cost of consumables for each isolation method.

	**dUC**	**SEC**	**UC + SEC**	**SEC + UC**
Isolation time	~2.5h	4-7h	6-8h	~3.5h
Nr Samples	1-44	1	1	1
Purity	++	+	++	++
Cost	+	++	+++	++

Table S5. One-way ANOVA of particle size from each method of EV isolation

Tukey's multiple comparisons test	Mean Diff.	95.00% CI of diff.	Below threshold?	Summary	Adjusted P Value	
dUC vs. SEC	-	-	No	ns	0.2062	A-
dUC vs. UC + SEC		-	No	ns	0.3716	A-
dUC vs. SEC + UC	-	-	No	ns	0.9627	A-
SEC vs. UC + SEC	13.88		Yes	**	0.0088	B-
SEC vs. SEC + UC	5.9	-	No	ns	0.4131	B-
	-	-	No	ns	0.1808	C-

Test details	Mean 1	Mean 2	Mean Diff.	SE of diff.	n1	n2	q	DF
dUC vs. SEC	125.5	133.2	-	3.718	5	5	2.921	16
dUC vs. UC + SEC	125.5	119.3		3.718	5	5	2.358	16
dUC vs. SEC + UC	125.5	127.3	-	3.718	5	5	0.6771	16
SEC vs. UC + SEC	133.2	119.3	13.88	3.718	5	5	5.28	16
SEC vs. SEC + UC	133.2	127.3	5.9	3.718	5	5	2.244	16
	119.3	127.3	-	3.718	5	5	3.036	16

Table S6. One-way ANOVA of particles/µg from each method of EV isolation

Tukey's multiple comparisons test	Mean Diff.	95.00% CI of diff.	Below threshold?	Summary	Adjusted P Value	
dUC vs. SEC	-	-	No	ns	0.5554	A-
dUC vs. SEC + UC	-1.30E+08	-	No	ns	0.6432	A-
dUC vs. UC + SEC	-2308999	-	No	ns	>0.9999	A-
SEC vs. SEC + UC	16400000	-	No	ns	0.9988	B-
SEC vs. UC + SEC	1.46E+08	-	No	ns	0.5677	B-
	1.30E+08	-	No	ns	0.6556	C-

Test details	Mean 1	Mean 2	Mean Diff.	SE of diff.	n1	n2	q	DF
dUC vs. SEC	2.29E+08	3.77E+08	-1.5E+08		5	5	1.888	16
dUC vs. SEC + UC	2.29E+08	3.61E+08	-1.3E+08		5	5	1.679	16
dUC vs. UC + SEC	2.29E+08	2.31E+08	-2.31E+06		5	5	0.02939	16
SEC vs. SEC + UC	3.77E+08	3.61E+08	-1.64E+07		5	5	0.2087	16
SEC vs. UC + SEC	3.77E+08	2.31E+08	1.46E+08	1.11E+08	5	5	1.858	16
	3.61E+08	2.31E+08	1.3E+08	1.11E+08	5	5	1.65	16

Table S7. One-way ANOVA of particles/mL FWB from each method of EV isolation

Tukey's multiple comparisons test	Mean Diff.	95.00% CI of diff.	Below threshold?	Summary	Adjusted P Value	
dUC vs. SEC	-2.40E+11	-	No	ns	0.5876	A-
dUC vs. UC + SEC	-2.20E+11	-	No	ns	0.653	A-
dUC vs. SEC + UC	-2.00E+08	-	No	ns	>0.9999	A-
SEC vs. UC + SEC	2.06E+10	-	No	ns	0.9995	B-
SEC vs. SEC + UC	2.40E+11	-	No	ns	0.5883	B-
	2.19E+11	-	No	ns	0.6537	C-

Test details	Mean 1	Mean 2	Mean Diff.	SE of diff.	n1	n2	q	DF
dUC vs. SEC	3.84E+11	6.24E+11	-2.40E+11		5	5	1.811	16
dUC vs. UC + SEC	3.84E+11	6.04E+11	-2.20E+11		5	5	1.656	16
dUC vs. SEC + UC	3.84E+11	3.84E+11	-2.00E+08		5	5	0.001508	16
SEC vs. UC + SEC	6.24E+11	6.04E+11	2.06E+10	1.88E+11	5	5	0.1553	16
SEC vs. SEC + UC	6.24E+11	3.84E+11	2.40E+11	1.88E+11	5	5	1.809	16
	6.04E+11	3.84E+11	2.19E+11	1.88E+11	5	5	1.654	16

Table S12. Differentially expressed proteins in cancer vs control

Gene symbol	logFC	adj.P.Val
HNRNPK	5.315308179	0.030815269
POTEF	5.105974434	0.015488242
IGKV2-28	4.772117592	0.009783439
ATP5F1A	4.015210553	0.002980438
HSP90B1	3.537961635	0.001604315
PAFAH1B2	3.266504146	0.030792863
RAB7A	3.231884697	0.000749313
PLP2	3.002729449	0.026284735
NAPRT	2.991427815	0.030792863
IPO7	2.960812916	0.082389673
HSPA8	2.892877793	0.000749313
PSMD13	2.843473969	0.009783439
APEH	2.831803542	0.055482915
PDLIM7	2.797611928	0.011556068
PSMD2	2.764053622	0.033150619
ENO1	2.704239518	0.060814071
CALM1	2.631854817	0.097706547
PSMA3	2.494231743	0.009783439
CAMP	2.46048189	0.097706547
ADD3	2.398150671	0.019113948
UGGT1	2.340659159	0.009031787
PSMD11	2.298650856	0.014256757
KPNB1	2.003528089	0.030792863
SOD2	1.20951053	0.097706547
TWF2	-1.866558792	0.097706547
ATP2A3	-1.895039375	0.052187277
ACTN1	-1.984443821	0.088824691
KNG1	-2.41111469	0.097706547
RAB14	-2.438988198	0.008295735
ADAM10	-2.489284914	0.007342118
LYPLA1	-2.884948651	0.00104798
ARPC1B	-2.993924623	0.00104798
APOE	-3.006117737	0.00083974

Table S13. Differentially expressed proteins in advanced vs non-advanced

Gene symbol	logFC	adj.P.Val
APOA1	4.252441195	0.000611
DMTN	-4.335353642	0.001181733
FBXL20	3.105967619	0.001181733
IGLC2	5.214923904	0.001181733
SEPTIN2	2.733504202	0.001182319
GPD2	-3.057340456	0.001285391
WDR77	-2.524110893	0.00518588
TMEM167A	-2.183034358	0.006214533
ACHE	-2.417877432	0.008456558
CAPNS1	-2.54272745	0.008456558
SERPINC1	2.215954399	0.008767701
MDH2	-2.409382057	0.008770068
CPT1A	3.435590497	0.010802746
TREML1	2.042181102	0.012757293
GSR	-2.357232443	0.019218077
APOC2	-1.723247364	0.019285121
HPRT1	-2.405648401	0.021119311
ATP1A1	-2.55128279	0.023489317
MPIG6B	2.18028275	0.023489317
MSN	-2.41054599	0.023489317
NRAS	-2.551904226	0.039226672
SAR1A	-2.260214788	0.039226672
ILK	2.349503129	0.043061068
HNRNPH1	2.328515977	0.043061068
TLN1	3.071988566	0.043849747
BCAM	-2.472484281	0.05105782
ATP7A	-3.682703771	0.053990725
PSMA2	1.849622496	0.060757677
CAP1	2.036431007	0.063668371
ACP1	-1.478054708	0.064634223
PSMD14	3.625782541	0.069856128
CAPZA1	1.984449698	0.069856128
CCT6A	1.860434249	0.072725829
HNRNPA1	-1.799715583	0.073425421
RAB11B	-2.034318244	0.082583356
PSMD1	-2.292161651	0.082756709
PRKACA	3.326473602	0.087569425
FN3K	-1.880773592	0.087569425
CIB1	-1.85809074	0.087569425
IGKV2D-24	2.308181759	0.087569425
PSMA5	2.323433325	0.095650297
